# miR-isomiRExp: a web-server for the analysis of expression of miRNA at the miRNA/isomiR levels

**DOI:** 10.1038/srep23700

**Published:** 2016-03-24

**Authors:** Li Guo, Jiafeng Yu, Tingming Liang, Quan Zou

**Affiliations:** 1 Department of Bioinformatics, School of Geographic and Biologic Information, Nanjing University of Posts and Telecommunications, Nanjing, 210023, China; 2 Shandong Provincial Key Laboratory of Functional Macromolecular Biophysics, Institute of Biophysics, Dezhou University, Dezhou, 253023, China; 3 Jiangsu Key Laboratory for Molecular and Medical Biotechnology, College of Life Science, Nanjing Normal University, Nanjing, 210023, China; 4 School of Computer Science and Technology, Tianjin University, Tianjin, 300072, China

## Abstract

MicroRNA (miRNA) locus has been found that can generate a series of varied isomiR sequences. Most studies always focus on determining miRNA level, however, the canonical miRNA sequence is only a specific member in the multiple isomiRs. Some studies have shown that isomiR sequences play versatile roles in biological progress, and the analysis and research should be simultaneously performed at the miRNA/isomiR levels. Based on the biological characteristics of miRNA and isomiR, we developed miR-isomiRExp to analyze expression pattern of miRNA at the miRNA/isomiR levels, provide insights into tracking miRNA/isomiR maturation and processing mechanisms, and reveal functional characteristics of miRNA/isomiR. Simultaneously, we also performed expression analysis of specific human diseases using public small RNA sequencing datasets based on the analysis platform, which may help in surveying the potential deregulated miRNA/isomiR expression profiles, especially sequence and function-related isomiRs for further interaction analysis and study. The miR-isomiRExp platform provides miRNA/isomiR expression patterns and more information to study deregulated miRNA loci and detailed isomiR sequences. This comprehensive analysis will enrich experimental miRNA studies. miR-isomiRExp is available at http://server.malab.cn/miRisomiRExp/.

Although microRNA (miRNA) has been extensively studied due to its flexible and crucial regulatory role, many literatures have validated that miRNA is not a single sequence, and each miRNA locus can generate a series of isomiRs with various sequences and expression patterns^1^,^2^. These multiple isomiR sequences, also known as miRNA variants or isoforms, were first observed during the analysis of high-throughput small RNA sequencing datasets^3^,^4^. Some of them are involved in diverged 5′ ends, which can lead to changes in functional “seed sequences” (nucleotides 2–8) or shifting of seed sequences (seed shifting). The change or shifting of seed sequences can further cause changes or shifting of target regions in sequence of the target mRNAs. Various isomiRs have been detected to have 3′ additional non-template nucleotides^5^,^6^, which in turn promotes events that may affect miRNA stability and target selection, ultimately affecting normal biological functions^6^,^7^. Although only a few isomiRs have been investigated in experiments because their canonical or annotated miRNA sequence is the main research target, some isomiR sequences, especially those isomiRs with 5′ variations and 3′ additions, have been determined to play important biological roles^8^,^9^,^10^,^11^,^12^,^13^.

IsomiR sequences are *in vivo* regulatory molecules, but most studies only focus on the canonical or annotated miRNA sequence. Indeed, the canonical miRNA is only one specific member of the family of isomiRs from a particular miRNA locus, and even is not abundantly expressed^2^,^5^. Most analyses have ignored the real regulatory sequences despite the fact that most of isomiRs are 3′ isomiRs with the same 5′ ends, and abundantly expressed 5′ isomiRs and isomiRs with 3′ additions should also be examined. More importantly, the direct and/or indirect interactions of these isomiRs with diverged sequences, particularly those based on miRNA gene clusters and families (clustered and homologous miRNAs loci always have close functional and sequence relationships), may have an important role in the coding-non-coding RNA regulatory network^14^. The phenomenon of multiple isomiRs significantly enriches miRNA study, especially for the potential complex and challenging interactions of isomiR-isomiR via diverse sequences with length divergence or sequence divergence or “seed shifting”. Simultaneously, the sequence diversity of multiple isomiRs is similar to miRNA sequence diversity across different animal species, suggesting that the phenomenon of multiple isomiRs may facilitate in identifying the optimal sequence involved in functional and evolutionary history^15^. Therefore, in miRNA studies, from the functional and evolutionary angles, isomiRs enrich the small RNA world as well as provide challenges that may further reveal details of the coding-non-coding RNA regulatory network.

Based on the current situation, it is thus necessary to establish a platform for the analysis of miRNA at the miRNA/isomiR levels that may further reveal and explore the small non-coding RNA world, particularly to better understand miRNA maturation and processing and functional characteristics. Some researchers have developed platforms to analyze miRNA at the isomiR level. For example, Sablok *et al*. reported an open-access web platform isomiRex, which identifies isomiRs and visualizes differentially expressed miRNAs^16^, Cheng *et al*. developed YM500 that is an integrated database for miRNA quantification, isomiR identification, arm switching discovery and normal miRNA prediction^17^,^18^, and Muller *et al*. reported an analytical tool that permits the deconvolution of miRNA heterogeneity and explores the functional role of isomiRs^19^. These platforms provide the possibility to analyze miRNA at the isomiR level, which largely contributes to miRNA/isomiR investigations, including further functional analysis of isomiRs. However, expression analysis of isomiRs may be insufficient for the in-depth analysis of expression of miRNA/isomiR. More importantly, in the regulatory network, expression and function of miRNAs/isomiRs do not only involve single but complex interactions, including miRNA/isomiR-miRNA/isomiR direct or indirect interactions. Herein, according to our previous studies and biological characteristics and association of miRNA and isomiR, especially for direct or indirect interaction of miRNAs, we developed the miR-isomiRExp platform and aimed to analyze the expression patterns of miRNAs at the miRNA/isomiR levels. Compared to other published relevant platforms, miR-isomiRExp contributes to tracking miRNA maturation and processing mechanisms, as well as reveals the functional characteristics of miRNA/isomiR.

## Materials and Methods

### The main analysis process

**Figure 1:**
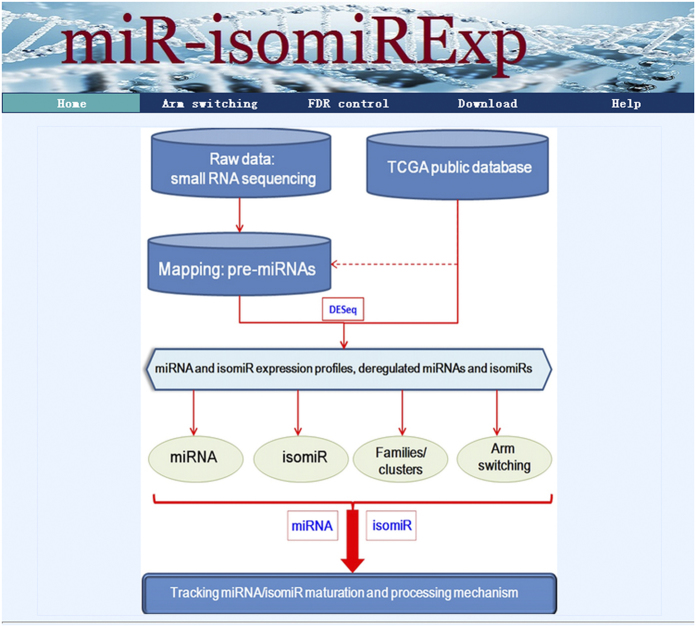
Flowchart for the analysis of miRNA at the miRNA and isomiR levels.

According to our recent studies^20^,^21^,^22^,^23^,^24^,^25^, the main analysis methods of miRNA/isomiR include the following steps (Fig. 1): (1) the candidate miRNA/isomiR expression profiles are obtained through mapping the genome and known precursor miRNA (pre-miRNA) sequences from the miRBase database (version 21.0, http://www.mirbase.org/)^26^ using the Bowtie software^27^. IsomiRs are obtained with ±5 nt along the canonical miRNA sequence that is obtained from the miRBase database; (2) Differentially expressed miRNA and isomiR species are first identified and screened using the DESeq package^28^ according to miRNA and isomiR expression profiles; (3) analysis at the miRNA levels: miRNA expression profiles, deregulated miRNA expression profiles, expression of specific grouped miRNAs, including clustered and homologous miRNAs with close physical distances and high sequence similarity respectively, sense-antisense miRNAs with reverse complementarity; (4) analysis at the isomiR levels based on miRNA locus: isomiR expression profiles and deregulated expression profiles are collected and analyzed based on its miRNA locus, and are further analyzed based on clustered and homologous miRNA loci; (5) analysis at the isomiR levels independent miRNA locus: isomiRs are estimated and analyzed at the isomiR levels based on the functional region of “seed sequences” without considering miRNA loci on the chromosomes; (6) the phenomenon of arm-switching is simultaneously analyzed at the isomiR levels because many miRNA genes have been determined to generate two mature miRNA products, thereby can be used to track and predict dynamic miRNA and isomiR expression profiles. A special miRNA class is defined according to classification and clustering the “seed sequences”, and further analysis is performed based on sequence and expression levels. The detailed sequences and enrichment levels are crucial in the analysis, and the novel homologous isomiRs are monitored at the isomiR levels.

### Reference database

According to the analysis pipeline, we further performed miRNA/isomiR analysis using small RNA sequencing datasets from public database of The Cancer Genome Atlas (TCGA) pilot project, including breast cancer, colon and rectal adenocarcinoma, head and neck squamous cell carcinoma, and other human diseases.

### Web-platform availability

The web server is written in Python, and source_codes can be downloaded from the web server (http://server.malab.cn/miRisomiRExp/).

## Results and Discussion

miRNAs have been extensively studied because of their crucial roles in multiple biological progresses, particularly pathological and physiological processes. However, the multiple isomiRs are rarely examined and in-depthly studied and most studies only focus on the canonical miRNA sequence. The canonical miRNA is only one of the specific isomiR sequences in the multiple isomiRs, and is not always the most dominant isomiR species. More importantly, isomiRs with sequence diversity and expression diversity engage in potential isomiR-isomiR and isomiR/isomiR-mRNA interactions in the coding-non-coding RNA regulatory network. Therefore, it is necessary to investigate the complex small RNA world based on these diverse isomiRs but with close functional relationships.

**Figure 2:**
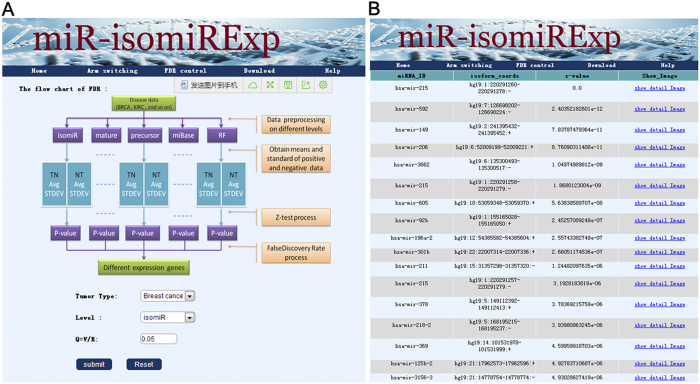
Differentially expressed miRNA/isomiR species in diseased samples. (**A**) The analysis pipeline; **(B**) The detailed screened result.

**Figure 3:**
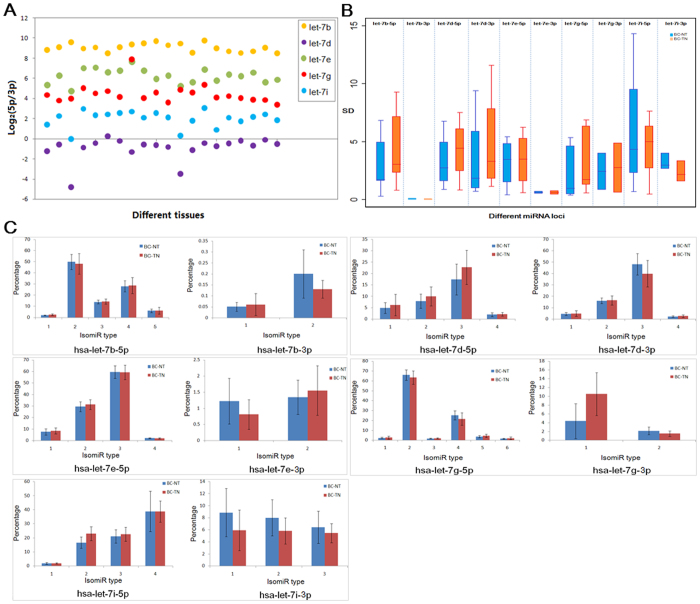
An example of the expression analysis of isomiR expression profiles in the let-7 gene family (including the isomiRs from miR-#-5p and miR-#-3p loci). (**A**) Scatter-plots of log_2_(5p/3p) in abundantly expressed let-7 members; (**B**) Box plots of let-7 members based on the 5p and 3p arms, respectively;(**C**) Expression of the dominant isomiR types in breast cancer (BC) tissues and normal tissues.

**Figure 4:**
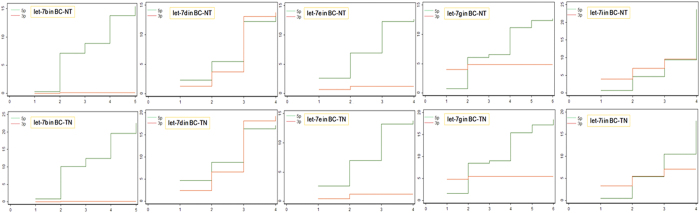
KS-test plots of isomiR distributions in the 5p and 3p arms, respectively, in the hsa-let-7 gene family.

Based on the findings of our previous studies, we propose a series of analysis for studying miRNAs at the miRNA/isomiR levels that may contribute to further revealing function and direct or indirect interactions of multiple isomiRs. Using the small RNA sequencing data annotated in the public TCGA database, a comprehensive analysis at the miRNA/isomiR levels was analyzed according to the pipeline of the miR-isomiRExp. Further experimental validation can be performed based on these collected target sequences (e.g., abnormally isomiRs in BC samples, http://server.malab.cn/miRisomiRExp/) (Fig. 2), miRNA and isomiR expression profiles in the specific samples, deregulated miRNA and isomiR expression profiles in diseased samples, potential disease-related miRNA and isomiR species, especially for those location and sequence related miRNA and isomiR species (Fig. 3). Moreover, the analysis of arm-switching is also presented in this study (Figs 3 and 4). Many miRNA genes can yield two mature miRNAs from the 5p and 3p arms in pre-miRNAs, and the maturation and expression processes may be related to clustered and homologous miRNAs. Therefore, the example justifies the need for the relevant analysis of homologous miRNAs in the hsa-let-7 gene family (some members are located in a gene cluster with close physical distances). The relevant results include the following: the scatter plot distribution of expression ratio between the 5p and 3p arms, expression distribution patterns, divergence expression of miRNAs using box and KS-test (Figs 3 and 4). These results contribute to the maturation and processing mechanism of miRNAs, particularly that involving dynamic switching during various spatiotemporal in the miRNA world.

Furthermore, we also performed the relevant functional analysis based on the screened 10 deregulated miRNA/isomiR loci in breast cancer, including let-7, miR-149, 125, 155, 15, 196, 200, 203, 204 and 21. Enriched results indicated that these miRNAs and isomiRs could contribute to multiple important biological processes and pathways, including regulation of transcription, chromatin modification, negative regulation of cell proliferation, MAPK signaling pathway, Wnt signaling pathway (Tables S1 and S2). Some of these relevant enriched pathways are consistent with reported cancer hallmarks (including cancer cell self-stimulate their own growth, resist cancer cell programmed cell death, multiply forever, invade local tissue and spread to distant organs, abnormal metabolic pathways, genome instability and inflammation) by Wang *et al*.^29^, indicating these relevant deregulated miRNAs/isomiRs may contribute to occurrence and development of breast cancer via regulating target mRNAs. Indeed, let-7 has been identified as an important miRNA in breast cancer^30^, and miR-21 is also a critical miRNA loci^31^. These results showed that the platform can provide deregulated miRNA/isomiR expression profiles for further functional analysis and experimental identification.

Compared to other platforms that analyze isomiRs, including isomiRex^16^ and YM500^17^,^18^, analytical tool^19^, miR-isomiRExp provides insights into resolving the mysteries of the small RNA world from the expression, evolutionary and functional levels based on miRNA/isomiR biological characteristics (Table 1). Firstly, at the expression level: isomiR expression profiles are analyzed from the miRNA loci and independent miRNA loci respectively, which can provide the differentially expressed isomiR expression profiles from various angles. Based on isomiRs yielded from the miRNA locus, we can analyze expression and function of these sequence and functional relevant isomiRs, and then track miRNA processing and maturation process. Independent miRNA loci, the whole isomiR expression profiles (across all the miRNA loci) are further analyzed, because many related miRNA loci (such as clustered and homologous miRNA loci) can yield the same or functional relevant isomiRs (have the same or relevant seed sequences but with different sequences and expression). IsomiRs can be further classified and clustered into functional groups based on their roles and potential interactions. Furthermore, the isomiR expression is further analyzed based on the phenomenon of arm switching. Many miRNA precursors, pre-miRNAs, can yield two kinds of abundant mature miRNAs from the miR-#-5p and miR-#-3p loci, although these isomiRs from 5p and 3p may be reverse compliment as well as miR-#-5p and miR-#-3p duplex. The potential isomiR-isomiR interaction further enriches the expression regulation in the small RNA world, although these isomiRs are generated from the same stem-loop structure and have the equal original amount of enrichment levels. Secondly, at the evolutionary level: the isomiR expression analysis is specifically analyzed from clustered and/or homologous miRNA gene loci, and these evolutionary or functional relevant isomiRs are easily clustered together to track their roles. Generally, these sequence or position-related miRNA loci yield sequence and/or functionally related isomiRs, and therefore these relevant multiple isomiRs should be not ignored, especially for its potential isomiR-isomiR interaction, including potential co-ordinated and restricted interactions. Simultaneously, some miRNA loci are sense and antisense miRNAs, and isomiRs from these relevant loci may also lead to isomiR-isomiR restricted interactions. These interesting and complex interactions may contribute to isomiR expression and function. Thirdly, at the functional level: functional analysis can be performed based on classified and clustered miRNA-class (these special miRNA-classes are classified and clustered using functional regions of isomiR sequences). Collectively, analysis of miRNAs using the miR-isomiRExp platform can provide a more comprehensive analysis of expression patterns of miRNAs and isomiRs, thereby generating profiles of isomiRs and potential miRNA-miRNA/isomiR-isomiR interactions based on sequence or location relationships. These results also contribute to tracking the miRNA/isomiR maturation and processing mechanisms, as well as revealing the expression and functional characteristics of miRNA/isomiR.

Taken together, the phenomenon of multiple isomiRs from the miRNA locus further enriches our understanding of miRNAs as well as future relevant studies in the wonderful small RNA world, especially those involved in RNA regulatory network. miR-isomiRExp provides more detailed expression results at the miRNA and isomiR levels, thereby promoting further experimental and functional studies on miRNA maturation and processing mechanism at the isomiR levels, and simultaneously contributing to our understanding of the functional relationships and characteristics among multiple homologous sequences in the coding-non-coding RNA networks.

## Additional Information

**How to cite this article**: Guo, L. *et al*. miR-isomiRExp: a web-server for the analysis of expression of miRNA at the miRNA/isomiR levels. *Sci. Rep.* **6**, 23700; doi: 10.1038/srep23700 (2016).

## Supplementary Material

Supplementary Information

## Figures and Tables

**Table 1:** The featured results provided by the miR-isomiRExp.

Analysis angle	The detailed results
Expression level	IsomiRs expression: based on the miRNA locus and independent miRNA loci, respectively
	IsomiRs yielded from 5p and 3p based on arm-switching
	Classified and clustered of isomiRs: miRNA-class
Evolutionary level	miRNA/isomiR expression based on clustered and/or homologous miRNAs (miRNA gene clusters and/or families)
Functional level	Further functional analysis based on miRNA-class at the isomiR level (functional clustering)
