# Supplementary material for: miR-isomiRExp: a web-server for the analysis of expression of miRNA at the miRNA/isomiR levels
Source: Sci Rep. 2016 Mar 24;6:23700. doi: 10.1038/srep23700 (PMC4806314; doi:10.1038/srep23700)
Supplement: Supplementary Information [file srep23700-s1.doc]

**miR-isomiRExp: a web-server for the analysis of expression of miRNA at the miRNA/isomiR levels**

Li Guo^a,*^, Jiafeng Yu^b^, Tingming Liang^c^ and Quan Zou^d,*^

^a^Department of Bioinformatics, School of Geographic and Biologic Information, Nanjing University of Posts and Telecommunications, Nanjing, 210023, China

^b^Shandong Provincial Key Laboratory of Functional Macromolecular Biophysics, Institute of Biophysics, Dezhou University, Dezhou, 253023,China

^c^Jiangsu Key Laboratory for Molecular and Medical Biotechnology, College of Life Science, Nanjing Normal University, Nanjing, 210023, China

^d^School of Computer Science and Technology, Tianjin University, Tianjin, 300072, China

*To whom correspondence should be addressed.

LG, [lguo@njupt.edu.cn](mailto:lguo@njupt.edu.cn); QZ, [zouquan@nclab.net](mailto:zouquan@nclab.net)

Table S1. The top 20 enriched biological processes based on selected 10 miRNA loci in breast cancer.

| **GO Term** | **Count** | **P-value** |
| --- | --- | --- |
| GO:0006355/regulation of transcription, DNA-dependent | 123 | 9.22E-128 |
| GO:0006350/transcription | 101 | 4.82E-92 |
| GO:0007165/signal transduction | 65 | 1.69E-38 |
| GO:0007275/development | 54 | 1.08E-36 |
| GO:0000122/negative regulation of transcription from RNA polymerase II promoter | 24 | 4.64E-35 |
| GO:0019941/modification-dependent protein catabolism | 31 | 1.76E-33 |
| GO:0006468/protein amino acid phosphorylation | 29 | 5.31E-28 |
| GO:0045944/positive regulation of transcription from RNA polymerase II promoter | 21 | 3.61E-27 |
| GO:0007399/nervous system development | 29 | 9.42E-26 |
| GO:0016568/chromatin modification | 18 | 2.39E-23 |
| GO:0030154/cell differentiation | 30 | 3.62E-21 |
| GO:0008285/negative regulation of cell proliferation | 17 | 1.32E-19 |
| GO:0007049/cell cycle | 24 | 3.59E-19 |
| GO:0045786/negative regulation of progression through cell cycle | 14 | 1.07E-18 |
| GO:0006470/protein amino acid dephosphorylation | 13 | 3.37E-18 |
| GO:0006814/sodium ion transport | 13 | 5.58E-18 |
| GO:0007155/cell adhesion | 21 | 7.81E-17 |
| GO:0006511/ubiquitin-dependent protein catabolism | 14 | 1.28E-16 |
| GO:0006811/ion transport | 20 | 3.51E-16 |
| GO:0006357/regulation of transcription from RNA polymerase II promoter | 18 | 6.27E-16 |

Relevant target mRNAs are regulated by at least 2 miRNAs/isomiRs, and target mRNAs are collected from TargetScan. GO enrichment is performed with CapitalBio Molecule Annotation System V4.0 (MAS, http://bioinfo.capitalbio.com/mas3/).

**Table S2.** The top 20 enriched KEGG pathways based on selected 10 miRNA loci in breast cancer.

| **Pathway** | **Count** | **P-value** |
| --- | --- | --- |
| MAPK signaling pathway | 28 | 9.38E |
| Wnt signaling pathway | 19 | 8.57E |
| Focal adhesion | 21 | 1.89E |
| Long-term potentiation | 14 | 9.94E |
| Axon guidance | 17 | 1.04E |
| TGF-beta signaling pathway | 13 | 1.49E |
| Renal cell carcinoma | 12 | 2.01E |
| Calcium signaling pathway | 17 | 3.09E |
| Gap junction | 12 | 5.26E |
| Glioma | 10 | 2.53E |
| Insulin signaling pathway | 13 | 5.15E |
| Melanogenesis | 11 | 2.33E |
| GnRH signaling pathway | 11 | 3.14E |
| Colorectal cancer | 10 | 3.23E |
| T cell receptor signaling pathway | 11 | 3.81E |
| ErbB signaling pathway | 10 | 4.55E |
| Prostate cancer | 10 | 5.08E |
| Non-small cell lung cancer | 8 | 1.38E |
| Chronic myeloid leukemia | 9 | 1.48E |
| B cell receptor signaling pathway | 9 | 1.48E |

Relevant target mRNAs are regulated by at least 2 miRNAs/isomiRs, and target mRNAs are collected from TargetScan. KEGG enrichment analysis is performed with CapitalBio Molecule Annotation System V4.0 (MAS, http://bioinfo.capitalbio.com/mas3/).
